# Tuberculous tenosynovitis of the Flexor Tendons of the hand and wrist: A case report and mini-review

**DOI:** 10.1016/j.amsu.2020.07.061

**Published:** 2020-08-07

**Authors:** Sitthiphong Suwannaphisit, Nakares Na Ranong

**Affiliations:** Department of Orthopaedic Surgery and Physical Medicine, Faculty of Medicine, Prince of Songkla University. 15 Karnjanavanich Road, Hat Yai, Songkhla, 90112, Thailand

**Keywords:** Tuberculosis, Tenosynovitis, Treatment outcome

## Abstract

Tuberculous tenosynovitis of hand and wrist is a rare disease but it is found sometimes, especially in TB-endemic areas. The clinical presentation is not specific, however, most patients present with painless swelling at the wrist and hand with limited range of motion, and nerve compression symptoms have been reported. The diagnosis of this conditions can be made from histopathology. Antituberculosis drugs are the mainstay treatment while surgery is controversial.

**Case presentation:**

We present the case of an 83-year-old Thai woman with no history of exposure to tuberculosis. She presented with swelling and mild pain at her right wrist and the fifth finger of her right hand for 3 months. Ultrasonography revealed tenosynovitis in the right hand and wrist. *Mycobacterium tuberculosis* was confirmed with tissue diagnosis after an open biopsy. 2-months regimens containing Isoniazid, Rifampicin, Pyrazinamide and Ethambutol/6-months of isoniazid and rifampicin treatment was successful without complications. We follower her up for 1 year, at which time she had returned to do normal daily activities. Her final DASH score was 10.8.

**Conclusion:**

Tuberculous tenosynovitis is rare, but still occasionally encountered, especially in TB-endemic areas. The challenge is that this condition is difficult to diagnose due to its clinically insidious onset and the presentation is not obviously specific. Laboratory analysis, imaging (MRI, ultrasonography) and microbiology are useful to help reach a diagnosis, but finally confirmation is from histopathology. The treatment mainstay is medical, but surgery may be required if conservative treatment fails or in late stages of the disease.

## Highlights

• Chronic painless swelling of the hand and wrist in TB endemic area should include TB tenosynovitis.
• Tissue pathology is the mainstays for diagnosis.
• The treatment mainstay is an anti-tuberculosis drugs regimen.

## 1 Introduction

Tuberculous tenosynovitis is an uncommon condition which affects the wrist and hand and accounts for 5% of musculoskeletal tuberculosis cases [1]. Tuberculous tenosynovitis usually causes chronic inflammation of the tendon and synovium with different symptoms and signs than acute flexor tenosynovitis. Clinical symptoms are usually not specific, with patients presenting with gradually increasing pain in the wrist, swelling around the wrist or hand, and limited range of motion. Such non-specific symptoms often lead to delayed diagnosis [2,3]. The most common method of reaching a conclusive diagnosis is histopathology. The characteristic feature of the granulomatous tissue in tuberculous tenosynovitis is caseous necrosis [4]. The main treatment is an antituberculosis drugs regimen, such as Isoniazid, Rifampicin, Pyrazinamide and Ethambutol, for 6–9 months [5]. Surgery is controversial, but recommended in patients who do not respond to antituberculosis drugs, or who have a limited range of motion or compression symptom [3,4].

### 1.1 Case report

**Fig. 1:**
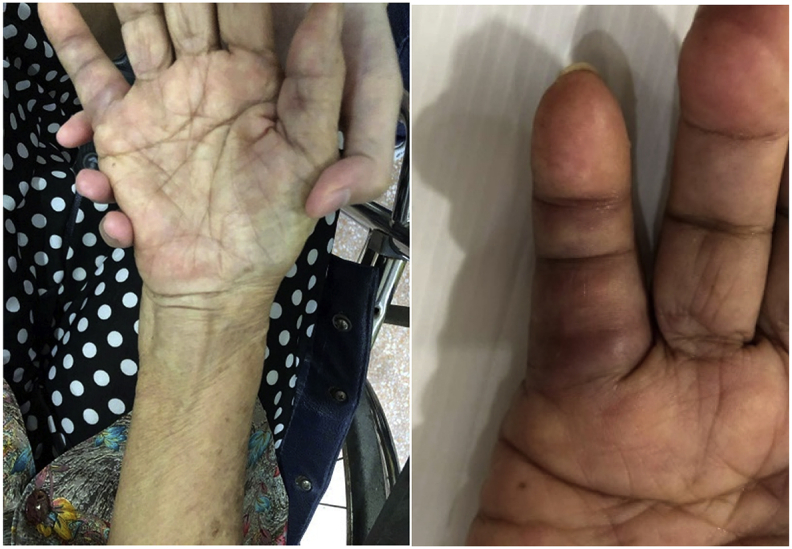
Pictures of our patient, showing swelling around volar surface of the right wrist and little finger.

**Fig. 2:**
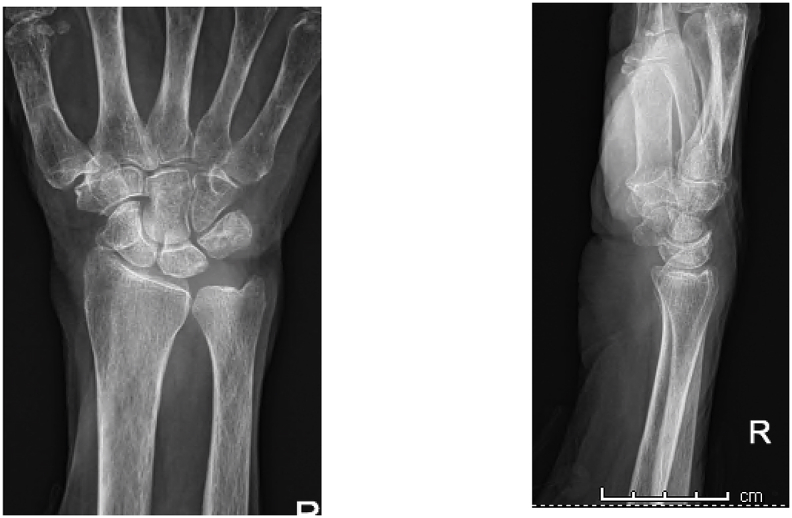
Anteroposterior and lateral radiographs views of the wrist showing no abbormalities.

**Fig. 3:**
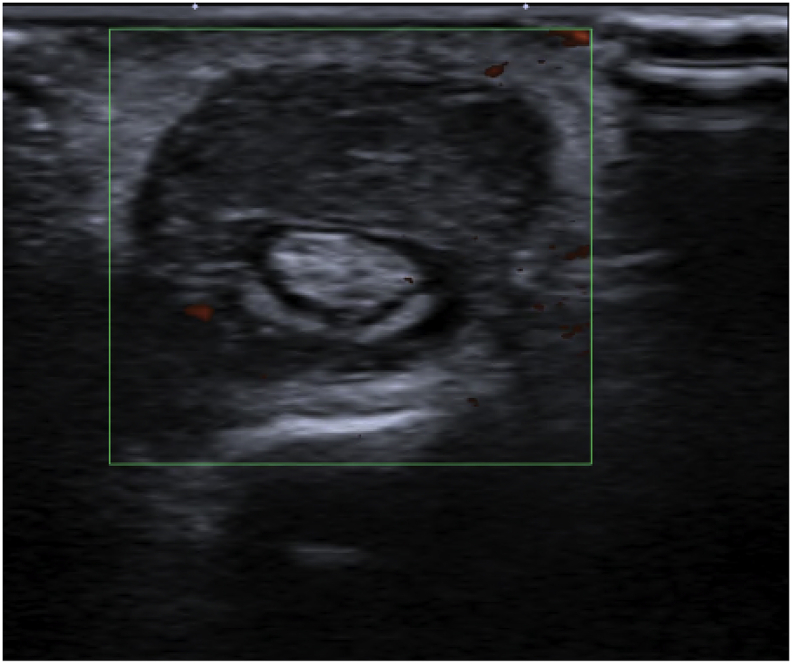
Transverse ultrasound view showing hypoechoic lesion volar to flexor digitorum tendon and tenosynovitis of 5th finger.

**Fig. 4:**
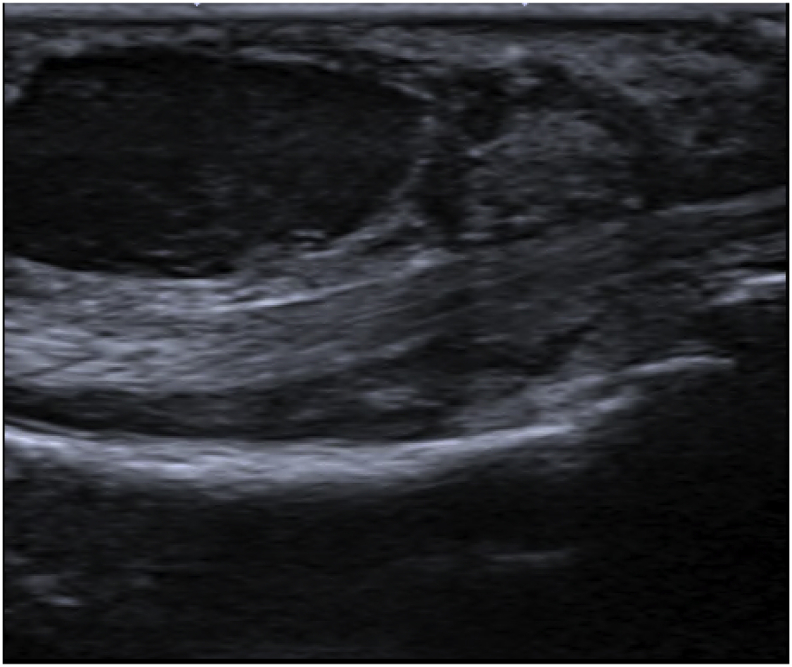
Longitudinal ultrasound view showing hypoechoic with cystic lesion volar to the flexor digitorum tendon of the 5th finger.

**Fig. 5:**
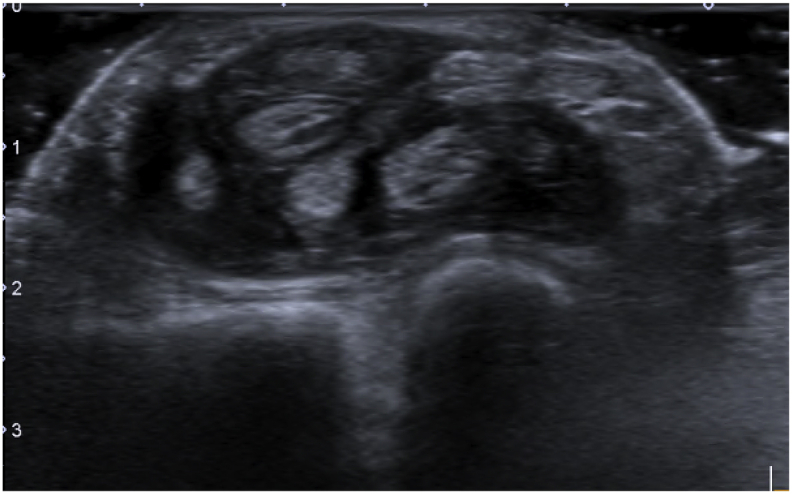
Transverse ultrasound view at wrist level showing thicke ning of the synovium and flexor tendon.

**Fig. 6:**
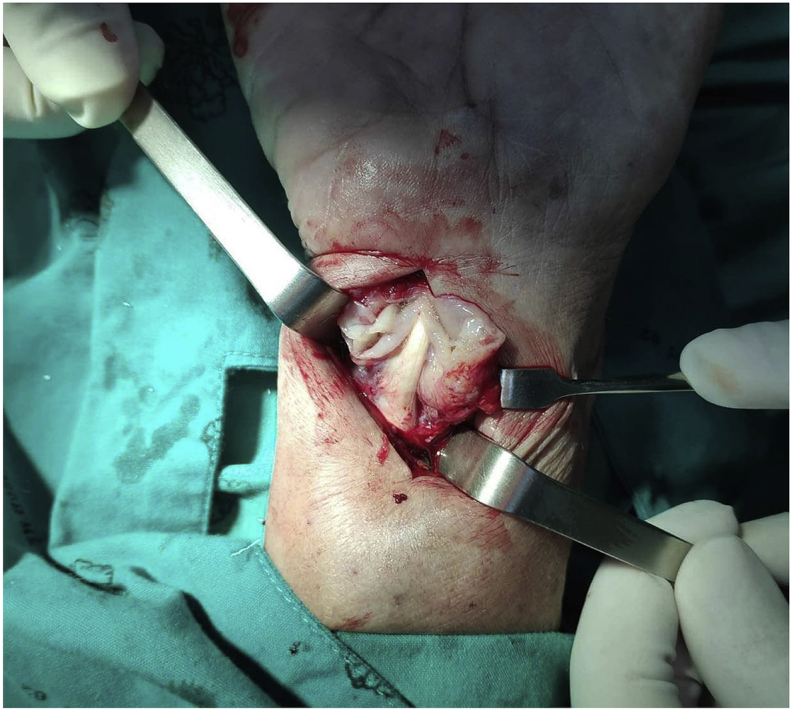
Intraoperative picture showing thickening of the synovium around the flexor tendon.

An 83-year-old Thai woman presented with gradual swelling around her right wrist for 3 months. She had no history of trauma around the wrist or fingers. She did not have diabetes mellitus, human immunodeficiency virus or exposure to tuberculosis. At the Orthopedic clinic a physical examination found mild pain and limited motion of her right wrist and the little finger, with some mild swelling at the wrist and the base of the little finger (Fig. 1). A DASH test gave a score of 59.2. Laboratory investigations including complete blood count, erythrocyte sedimentation rate, and C-reactive protein were all in normal levels. A chest radiograph was normal. Plain radiographs of the wrist showed generalized osteopenia with some sclerotic change, with no osteolytic lesions (Fig. 2). Ultrasonography indicated chronic tenosynovitis of the flexor digitorum tendons with more localized fluid at the distal forearm and little finger. Two oval-shaped homogenous hypoechoic cysts on the volar aspect of the hypoechoic flexor tendon of the right 5th finger suggested chronic tenosynovitis of the 5th flexor tendon compatible with tuberculosis (Fig. 3, Fig. 4, Fig. 5). We did an open biopsy of the wrist and little finger, and found thick yellowish synovial encasing the flexor tendon with no pus or purulence (Fig. 6). At the base of the little finger, where some pus was present; after manipulation to loosen the tendon at the base of the finger, the pus was removed and cultured was obtained. The culture showed caseous granulomatous chronic inflammation compatible with tuberculosis and acid-fast gram stain positive and PCR for TB was positive for *M.tuberculosis* complex in both the fluid from the base of the right little finger and tissue from the right wrist. An infectious disease clinician was consulted for further evaluation of the patient. Sputum morning acid-fast gram stain was done and was negative for 3 consecutive days. She was treated with a standard four-drug regimen (isoniazid, rifampicin, ethambutol and pyrazinamide) for 2 months and the isoniazid and rifampicin for an additional 6 months, during which time her clinical condition slowly improved, with decreased swelling and improved wrist and fingers motion within normal ranges with no side effects from the anti-tuberculosis drugs. The last follow up at 1 year, at which time she was satisfied with her condition with a final DASH score of 10.8.

## 2 Discussion

Extrapulmonary tuberculosis in the United States accounts for only about 20% of all TB cases, with osteoarticular TB occurring in about 2–5% of the cases [1,5]. The most common locations for osteoarticular TB are the spine, hip and knee. Hand involvement is rare, occurring in only about 10% of all osteoarticular lesions, with most of these found in the flexor side and ulnar border [3,6]. The pathogenesis of Tuberculous tenosynovitis is commonly from hematogenous spread from the lungs, with other sources the lymph nodes, bone, genitourinary system or direct inoculation. The risk factors for this type of disease are male, dominant hand, overuse of the hand, old age, low socioeconomic status, malnutrition and immunosuppression [1]. Our patient had several of these factors; although she was female rather than male, she was old, with a history of overuse of her hand, low socioeconomic status as she used to be farmer, and susceptible to direct inoculation as Thailand is endemic for TB. Tuberculous tenosynovitis has 3 stages: Hygromatous stage there is serous exudate in tendon sheath and tendon is normal, next serofibrinosus stage granulation tissue along tendon and finally fungoid stage caseous, abscess formation and tendon rupture [3,6,7]. There are some classic presentation of tuberculous tenosynovitis first, the compound palmar ganglion: this is the most common presentation in tuberculous tenosynovitis there is painless swelling proximal and distal to carpal tunnel and most involved the flexor sheath of the little finger and extended to the ulnar bursa moreover rice bodies are frequently seen with this type. Second, the sausage digit: this presentation is usually seen as single digit of the index, middle or ring fingers. Third, carpal tunnel syndrome: patient present with classic carpal tunnel syndrome with volar wrist or distal forearm swelling [3]. However due to this condition is rare and clinical are can be found in other condition that may be leading to misdiagnosis [7]. In Thailand, tuberculous tenosynovitis should be in the differential diagnosis when a patient presents with chronic wrist or hand swelling and limited range of motion, especially in areas endemic for tuberculosis. Laboratory investigations such as complete blood count, erythrocyte sedimentation rate, or C-reactive protein may be normal or elevated in such cases, but these are not specific tests for tuberculous tenosynovitis and the condition cannot be excluded when these tests are normal [5]. Chest radiography in musculoskeletal tuberculosis mostly reveal normal.Therefore, a normal chest x-ray cannot exclude a diagnosis of tuberculous tenosynovitis [5] and radiographic bone films may not show bony abnormalities [1]. Ultrasonography is a useful diagnostic tool for evaluating a situation suspicious of tenosynovitis, as US can evaluate synovium thickening, peritendinous effusion, pus collection and extensions of a lesion [[7], [8], [9]]. Magnetic resonance imaging (MRI) is more sensitive and specific than ultrasonography, however it is not available in some countries [7]. In our case, chest and bone radiographs did not show any abnormalities while ultrasonography revealed flexor tenosynovitis which was highly suspicious of chronic tenosynovitis so we did not need MRI. Although tenosynovitis can be detected using ultrasonography and MRI. Microbiology studies are insufficient because AFB can be positive in synovial fluid 10%, synovial tissue 20% and regional lymph nodes 30% and cultures in Lowenstein and BACTEC media are usually negative from extrapulmonary tuberculosis, and therefore, negative findings cannot exclude this condition. Histopathology is used to confirm the diagnosis in tuberculous tenosynovitis, with characteristic findings of caseous granulomas, tuberculosis bacillus, and multinucleated Langerhans giant cells [1,4]. Our case culture and AFB were done and showed positive results and histopathology showed granulomatous inflammation.

Treatment of tuberculous tenosynovitis consists of a standard anti-tuberculosis four-drug regimen (isoniazid, rifampicin, pyrazinamide and ethambutol) for 2 months followed by isoniazid and rifampicin for 4 months. Some studies [5,10] recommend a longer treatment course, up to 18 months. In our case, we reviewed the previous literature on treatment of TB tenosynovitis of the wrist and hand (Table 1). Anti-tuberculosis drugs should be started as soon as possible after diagnosis and completing the full course is essential to prevent recurrence and in case of resistant strains. Some studies [4] have found 75% success rate with anti-tuberculosis drugs without surgical intervention and a return to full activity in an average of 5 months. In cases in which surgical treatment is required, debridement should be done when antituberculosis drugs fail, or in stage 2 or 3 disease [3,6]. In our patient was performed a tenosynovectomy and prescribed IRZE for 2 months and IR for 6 months, after which she recovered fully with no complications from either the anti-tuberculosis drugs or surgical debridement.

**Table 1:** Previous studies on tuberculosis of the hand and wrist.

First Author (Year)	Article type	Area	Treatment	Result	Complication
Sbai MA (2015)	Case report	Tunisia	Synovectomy + 2IRZE/6IR	Follow up 3 years, good outcome	None
Bayram S (2016)	Case report	Turkey	Synovectomy + Anti-TB (12 months)	Follow up 1-year, good outcome	None
Kabakas F (2016)	Retrospective (13 cases)	Turkey	3IRZE/6IR	9 months	None
Chan E (2017)	Review article	USA	2IRZE/4IR	N/A	N/A
Hogan JI (2017)	Review article	USA	2IRZE/4IR	6–8 months, good outcome	Abscess, osteomyelitis require surgery
Cohen-Tanuki S (2018)	Case report	USA	Synovectomy + 2IRZE/4IR	6 months, good outcome	None

## 3 Conclusion

Tuberculous tenosynovitis, although rare, is still occasionally encountered, especially in TB endemic areas. The first challenge is difficulty in diagnosing this condition, due to clinically insidious onset and non-obvious presentations. Laboratory analysis, imaging (MRI, ultrasonography, plain radiograph) and microbiology are useful to help with the diagnosis, but final confirmation is usually from histopathology. The treatment mainstay is conservative with anti-TB drugs, but surgery may be required in more difficult or late-stage situations.

## Please state any sources of funding for your research

No funding was involved regarding this case report.

## Ethical approval

The present study was approved by the Prince of Songkla University Institutional Review Board, Faculty of Medicine, Songklanagarind Hospital, Prince of Songkla University (IRB number REC 63-271-11-1).

## Consent

Written informed consent was obtained from the patient for publication of this case report and accompanying images. A copy of the written consent is available for review by the Editor-in-Chief of this journal on request.

## Author contribution

SS, first author, performed the intervention. Both authors, SS and NN, contributed to manuscript. Both authors have read and agreed with the manuscript.

## Registration of research studies

None.

## Declaration of competing interest

No conflicts of interest.
